# Comprehensive analysis of the tumor immune micro-environment in non-small cell lung cancer for efficacy of checkpoint inhibitor

**DOI:** 10.1038/s41598-018-32855-8

**Published:** 2018-10-01

**Authors:** Jeong-Sun Seo, Ahreum Kim, Jong-Yeon Shin, Young Tae Kim

**Affiliations:** 1 Precision Medicine Center, Seoul National University Bundang Hospital, Seongnamsi, 13605 Korea; 2 Genomic Medicine Institute, Medical Research Center, Seoul National University, Seoul, 03080 Republic of Korea; 3 Department of Biomedical Sciences, Seoul National University College of Medicine, Seoul, 03080 Republic of Korea; 4 Seoul National University Cancer Research Institute, Seoul, Republic of Korea; 5 Department of Thoracic and Cardiovascular Surgery, Seoul National University Hospital, Seoul, 03080 Korea; 6 Macrogen Inc., Seoul, 08511 Korea

## Abstract

Characterizing the molecular immune subtype and micro-environment of lung cancer is necessary to understand immunogenic interactions between infiltrating immune and stromal cells, and how tumor cells overcome immune checkpoint blockades. This study seeks to identify computational methodologies for subtyping gene expression-based tumor-immune micro-environment interactions, which differentiate non-small cell lung cancer (NSCLC) into immune-defective and immune-competent subtypes. Here, 101 lung squamous cell carcinomas (LUSCs) and 87 lung adenocarcinomas (LUADs) tumor samples have been analyzed. Several micro-environmental factors differentially induce LUAD or LUSC immune subtypes, as well as immune checkpoint expression. In particular, tumor-associated macrophages (TAMs) are key immune cells play a vital role in inflammation and cancer micro-environments of LUSCs; whereas, regulatory B cells are immunosuppressive and tumorigenic in LUADs. Additionally, cytolytic activity upon CD8^+^ T cell activation is decreased by the abundance of B cells and macrophages in immune-competent subtypes. Therefore, identifying immune subtypes in lung cancer and their impact on tumor micro-environment will lead to clinical tools for assessing LUADs and LUSCs in patients, as well as maximize the efficacy of immune checkpoint inhibitors.

## Introduction

Lung cancer is the most common cancer diagnosis and cause of death in Korea. Lung squamous cell carcinoma (LUSC) and lung adenocarcinoma (LUAD) are two major subtypes of non-small cell lung cancer (NSCLC), which, together, account for approximately 60% of all lung cancer diagnoses in Korea^1,2^. These subtypes exhibit significant differences in molecular organization and activity^3^.

Patients with LUSCs tend to be smokers and have a *TP53* mutation; whereas, LUAD patients have several key mutations in *EGFR*, *KRAS*, *NRAS*, *BRAF*, *PIK3CA*, *MET*, and *CTNNB1* genes^4,5^. Although the somatic mutations in each NSCLC subtype have been well-characterized, the fundamental differences in NSCLC micro-environment and its interaction with two major types of NSCLC have not yet been comprehensively explored. Similarly, the molecular mechanisms involved in pathogenicity have mainly been opaque^6,7^. Thus, it is important to characterize the genomic mutations and risk factors involved in LUAD and LUSC, since these factors can impact immunity and tumor micro-environment, depending on cancer type^8^.

In previous studies, the mutational burden and neo-antigen load were shown to be associated with favorable responses to immunotherapy in specific patients; however, cataloging mutations load alone is not a sufficient predictor of responsiveness to immunotherapy^9–11^. Moreover, multiple changes in tumor micro-environments, as well as emergent immunogenic mechanisms, enable resistance to immune checkpoint inhibitors. For this reason, the single-agent anti PD-1/PD-L1 is of minimal clinical benefit to patients^12–14^. Importantly, the micro-environment and immune cells infiltrating the tumor are unique to each cancer type; thus, studying such conditions for each cancer type will be crucial in elucidating immune checkpoint blockades such as the PD-1 inhibitor^15,16^.

Therefore, this study focuses on utilizing computational methodologies to characterize gene expression in immune subtypes and identify fundamental differences in the micro-environmental signatures of LUADs and LUSCs. With this information, predictive biomarkers of infiltrating immune cells and the tumor microenvironments surrounding NSCLC subtypes could be developed to identify patients who will be receptive to immune therapies.

## Results

### Identification of immune subtypes in response to LUADs

A total of 87 LUAD samples and 77 matched noncancer controls were analyzed to identify the gene expression responsible for LUAD immune subtypes, using a method previously reported by Seo *et al*.^17^. In particular, principal component analysis (PCA) algorithms were utilized to evaluate 1,000 of the most variable genes and perform unsupervised hierarchical clustering. Here, the LUADs and noncancer control clusters were sufficiently separated, while TCGA LUADs (*n* = 451) and TCGA noncancer controls (*n* = 49) were similarly clustered, with a 95% confidence interval (Supplementary Fig. S1).

Additional unsupervised k-means (*n* = 3) hierarchical clustering between LUADs and noncancer control samples provided three clusters of two LUAD samples and one mixture of LUADs and noncancer control samples. Similarly, TCGA LUAD cohorts had the same pattern, with three distinguishable clusters of two TCGA LUAD clusters and one mixture of LUADs and noncancer control samples (Supplementary Fig. S2).

**Figure 1:**
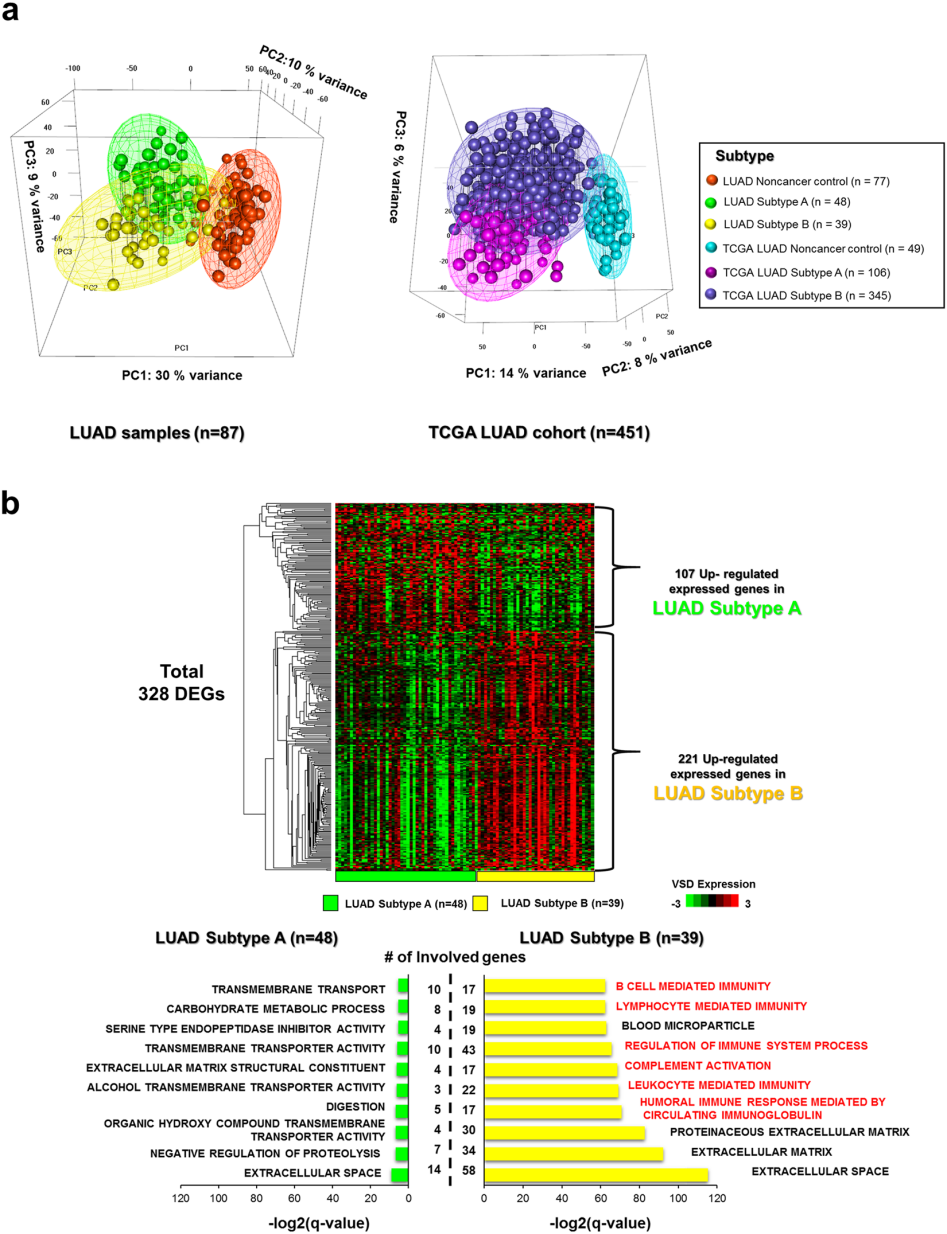
Transcriptomic analyses for immune subtypes in LUADs. (**a**) Subtypes A, B, and noncancer control were distinguishable by the first three PCs of the top 1,000 most variable genes, across all samples in LUADs (*n* = 87) and TCGA LUADs (*n* = 451). The meshes containing each subtype (A, B, and noncancer control) were drawn by performing unsupervised hierarchical clustering and k-means clustering (k-means = 3), with 95% confidence interval ellipsoids in LUAD and TCGA LUAD samples. (**b**) The VSD-normalized expression of differentially expressed genes in Subtypes A and B of LUADs are illustrated in the heatmap. Top 10 GO gene sets in either Subtype A or B were selected based on the rank of enrichment –log_2_(q-value) for the pathway and matched significance criteria (p-value < 0.05 and FDR q-value < 0.1). A two-color scale was used, with green indicating low expression values and red representing highly expressed genes.

We defined cluster 2 as Subtype A and the combination of clusters 1 and 3 as Subtype B, for both LUADs and TCGA LUADs. The LUADs and TCGA LUADs in the cluster 3, which had both noncancer control and a few LUAD samples, seemed to be normal like cancer since the samples were grouped with the majority of noncancer control samples and would have the high portion of infiltrating immune cells. The infiltrating immune cells in the cancer samples tend to affect the tumor purity, fraction of cancerous cells, so we assumed that the LUADs in this cluster might be one of cancer types, which is highly immunologically competent subtype and has the higher portion of infiltrating immune cells. Therefore, we defined the LUADs and TCGA LUADs in this cluster 3 as the immune competent subtype (Subtype B), and the LUADs in the other two clusters indicated as the immune competent subtype (Subtype B) and immune deficient subtype (Subtype A) via the enrichment of Gene Ontology gene sets with differentially expressed genes. Also, additional PCA plots with LUAD and TCGA LUAD samples revealed the separation between Subtype A and Subtype B at the 95% confidence interval (Fig. 1a).

Different compositions of patient populations were assigned to each subtype, since sample sizes for reference patients, such as those with the TCGA LUAD noncancer control (*n* = 49), were too small to normalize expression levels in TCGA LUAD samples when compared to LUAD samples and their matched noncancer control sample sizes. Thus, the expression level of LUAD samples could be decreased and normalized in accordance with the noncancer control sample than TCGA LUAD samples^18^.

Differentially expressed genes (DEGs) in each subtype were investigated in LUAD and TCGA LUAD cohorts (DESeq: *P* < 0.05 and FDR < 0.1; Fig. 1b and Supplementary Fig. S3). There were 107 upregulated expressed genes in LUAD Subtype A, while the same genes were downregulated in LUAD Subtype B. These 107 genes were enriched in the extracellular space, negatively regulated proteolysis, and transmembrane transport.

The 221 upregulated expressed genes in LUAD Subtype B were downregulated in LUAD Subtype A. These genes were closely associated with immune gene sets such as those involved in humoral immune responses mediated by circulating immunoglobulin and leukocyte-mediated immunity. The DEGs of Subtype A in the TCGA cohort were enriched in gene clusters involved in neuron and synapse activity, but not in LUAD Subtype A. However, DEGs of the TGGA LUAD Subtype B cohort were enriched in similar immune-related gene sets of LUAD Subtype B.

Interestingly, the enrichment log(q-value) of gene sets of Subtype A in both cohorts were smaller than those of Subtype B. This indicated that Subtype B was more strongly associated with immune gene clusters than Subtype A. Furthermore, the 89 overlapping upregulated genes in LUADs and TCGA LUADs were mostly enriched in immune gene clusters such as humoral immune response and B cell-mediated immune cells; therefore, Subtype B in LUAD and TCGA LUAD cohorts was confirmed to be immunologically associated, similar to other previously elucidated immune competent subtypes^17^ (Supplementary Fig. S4).

Subtype A, on the other hand, was largely immune-suppressive subtype; whereas, the immune profiles of Subtype B (immune-competent) followed a similar pattern as the immune subtype of head and neck squamous cell carcinoma (HNSCC), which exhibits upregulation of immune-related genes and enhanced tumor micro-environment. This suggests that patients with these subtypes would also benefit from immunotherapy^19^.

### Estimation of immune and micro-environmental factors between subtypes

In order to identify the impacts of immunogenic and micro-environmental factors – such as the immune and stromal score, cytolytic score, and tumor purity, as well as the abundance of immune cells on LUAD subtypes–were estimated via previously reported methods^20,21^. The stromal score, which designated to capture the infiltrating stromal cells in tumor tissue, was highly correlated with the immune score indicated the infiltration of immune cells in LUAD samples, but there was no significant difference in correlations between stromal and immune scores among cohorts (Subtype A: Pearson’s *r* = 0.86; Subtype B Pearson’s *r* = 0.86; TCGA LUAD Subtype A: Pearson’s *r* = 0.75; TCGA LUAD Subtype A: Pearson’s *r* = 0.64). Only noncancer control samples had low correlation between Subtypes A and B (LUAD Noncancer control: Pearson’s *r* = 0.35; TCGA LUAD Noncancer control: Pearson’s *r* = 0.32; Supplementary Fig. S5).

**Figure 2:**
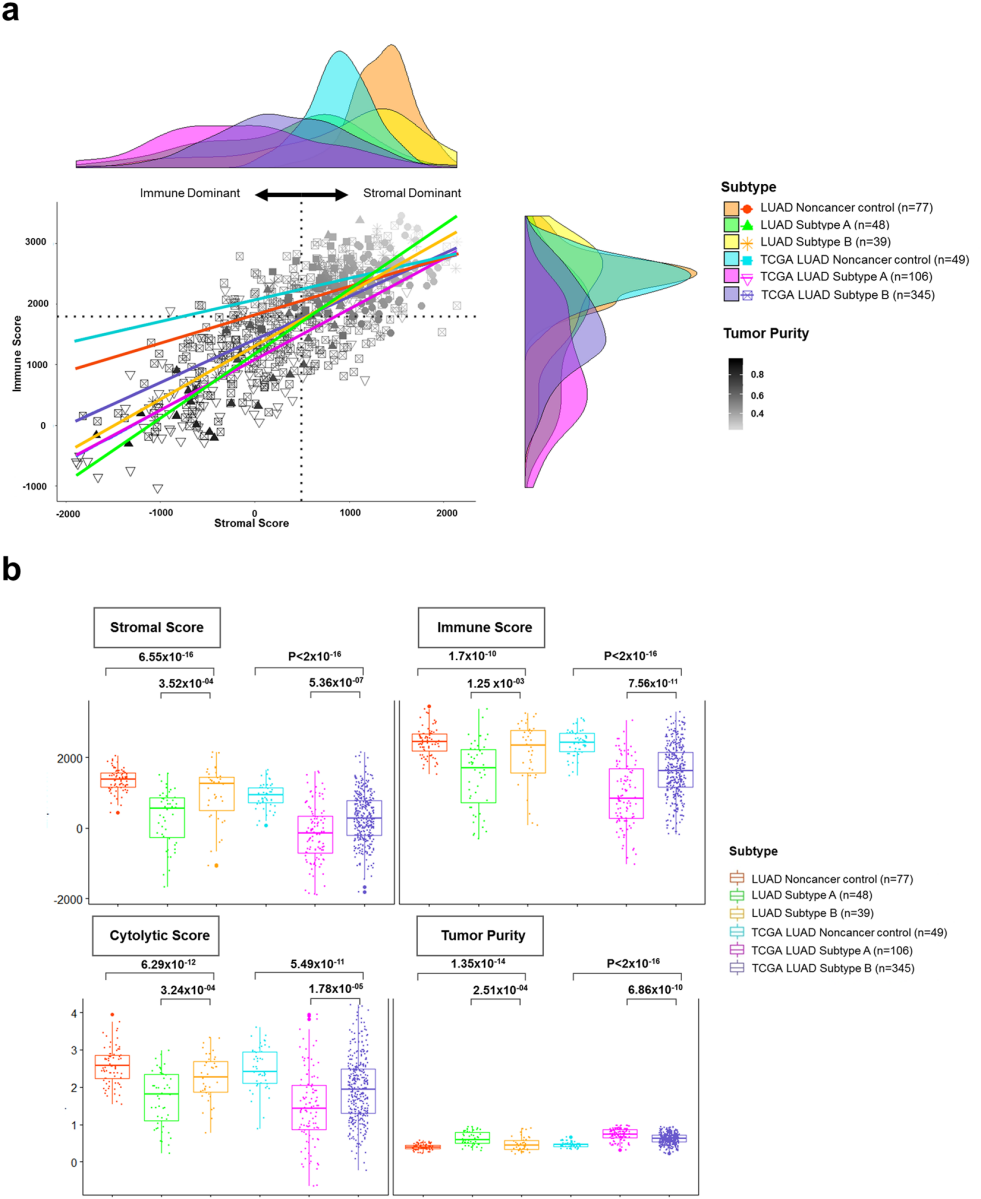
Estimation of micro-environmental factors between subtypes in LUADs. (**a**) Scatterplots and marginal density plots between stromal and immune scores are illustrated. The tumor purity was used as the color grading for sample points, and its index is shown on the color bar at the bottom right of the plot. The median score is indicated by dashed lines under the horizontal (x) and vertical (y) axis. (**b**) Several generated micro-environmental factors (stromal, immune, cytolytic (CYT) score, and tumor purity) were compared between subtypes and each p-value was indicated by an unpaired Student’s *t* test based on the sample distribution test.

The tumor purity was relatively low at the stromal dominant position, and only noncancer control samples were densely packed at this position (Fig. 2a). Overall, the plot and correlation table explained the fact that the stromal and immune cells were strongly and directly associated with cancer cells, regardless of subtype, in both cohorts. Moreover, the complex of stromal, immune, and cancer cells promotes tumor growth and provides a favorable micro-environment for a pro-tumorigenic immune subtype^22,23^. Here, several tumor micro-environment factors–including stromal, immune, cytolytic score, and tumor purity–were compared between subtypes. All tumor micro-environment factors were statistically different between subtypes in both cohorts (Fig. 2b).

Stromal, immune, and cytolytic scores for Subtype B were much higher than for Subtype A, while the tumor purity in Subtype B was much less than in Subtype A (LUAD Cohort: *P* = 2.51 × 10^−4^ via unpaired Student’s *t* test; TCGA LUAD Cohort: *P* = 6.86 × 10^−10^ via unpaired Student’s *t* test). Additionally, infiltrating immune cells such as B cells, CD8^+^ T cells, and dendritic cells were more prevalent in response to Subtype B than Subtype A in both cohorts (LUAD Cohort: *P*_*B cells*_ = 2.23 × 10^−2^; *P*_*CD8*+ *T cells*_ = 2.63 × 10^−03^, *P*_dendritic cells_ = 6.94 × 10^−05^ via unpaired Student’s *t* test; TCGA LUAD cohort: *P*_*B cells*_ = 3.76 × 10^−13^, *P*_*CD8*+ *T cells*_ = 1.30 × 10^−2^, *P*_*dendritic cells*_ = 6.22 × 10^−4^ via unpaired Student’s *t* test; Supplementary Fig. S6).

Interestingly, CD8^+^ T cells were abundant in the LUAD and TCGA LUAD noncancer control samples, which also had a high stromal score and low tumor purity compared to LUAD and TCGA LUAD cancers. Therefore, the abundance of CD8^+^ T cells in normal tissue indicated increased expression of inflammatory markers, and thereby represented an intermediate state between normal to cancerous tissue. This result is in accordance with previous findings^24^.

There was no significant difference in the correlation coefficient between six types of immune cells in response to Subtypes A and B in both LUAD and TCGA LUAD cohorts; however, B cells were not significantly correlated with other immune cells, and CD 4^+^ T cells and CD8^+^ T cells were similarly indirectly related to the prevalence of such cell subtypes in both cohorts (Supplementary Fig. S7).

### The micro-environmental signature and immune checkpoints of LUADs

**Figure 3:**
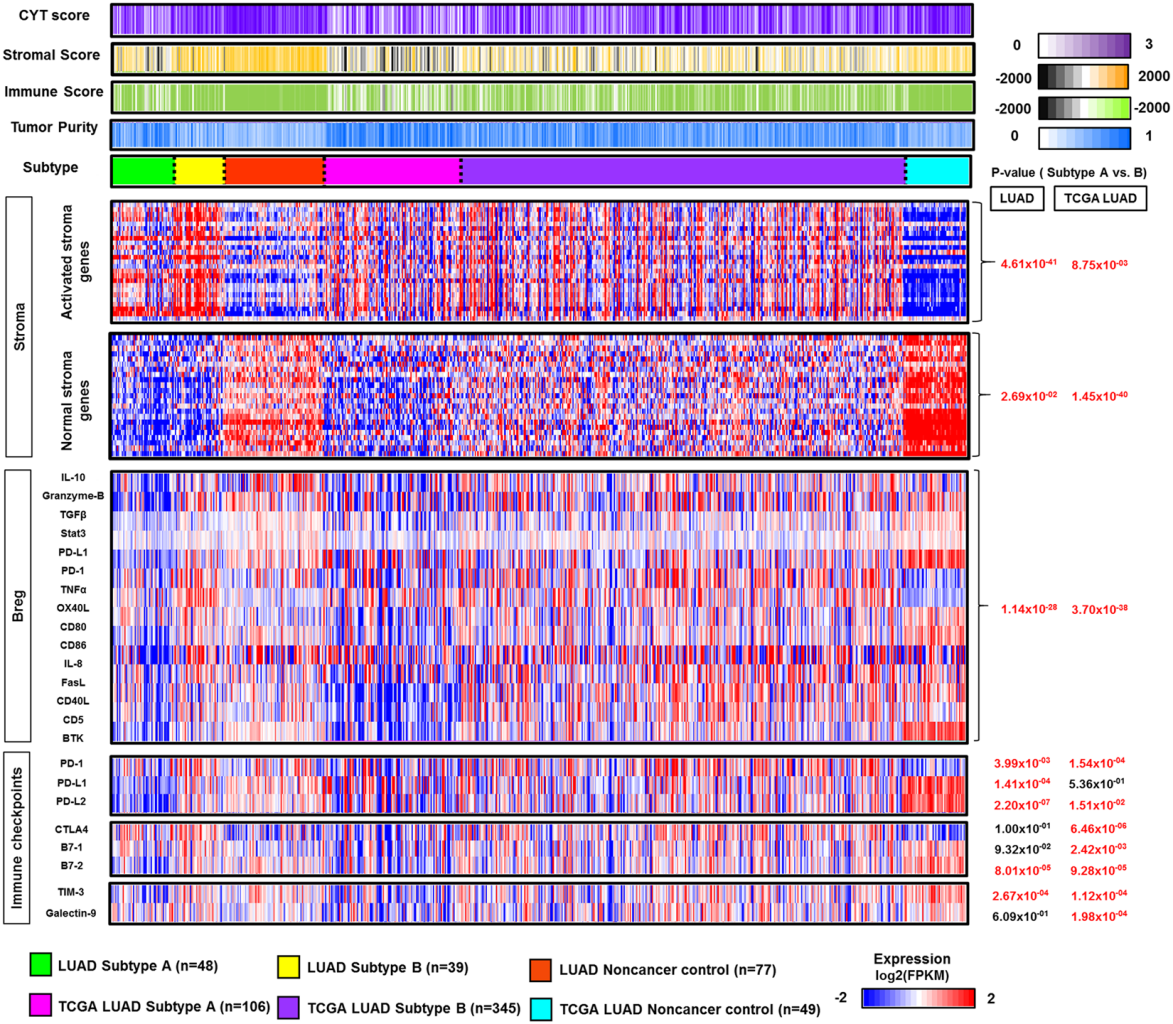
Micro-environmental signature and immune checkpoint expression in LUAD subtypes. The heatmap depicts the level of the tumor micro-environmental factor (cytolytic (CYT) score, stromal score, immune score, tumor purity), as well as the expression of activated stromal and normal stromal genes, regulatory B representative genes, and immune checkpoint genes in LUAD and TCGA LUAD subtypes (Subtype A, B, and noncancer control). A comparison of median-centered expression (log_2_fpkm) of each factor was performed between subtypes, and the p-value was indicated by an unpaired Student’s *t* test based on the normality. A two-color scale was used, with blue indicating low expression values and red representing highly expressed genes.

The expression of activated stroma and normal stromal genes and immunologic factors such as representative regulatory B cell genes and immune checkpoint genes were compared between subtypes in the LUAD and TCGA LUAD cohorts (Fig. 3). The genes of activated stroma and regulatory B cells in Subtype B had greater expression than those of Subtype A in both cohorts (LUAD cohort: *P*_*activated stromal genes*_ = 4.61 × 10^−41^, *P*_*B reg*_ = 1.14 × 10^−28^ via unpaired Student’s *t* test; TCGA LUAD cohort: *P*_*activated stromal genes*_ = 8.75 × 10^−03^, *P*_*B reg*_ = 3.70 × 10^−38^ via unpaired Student’s *t* test). Similarly, normal stromal genes were expressed with greater volume in Subtype B than Subtype A (LUAD cohort: *P*_*normal stromal genes*_ = 2.69 × 10^−2^ via unpaired Student’s *t* test; TCGA LUAD cohort: *P*_*normal stromal genes*_ = 1.45 × 10^−40^ via unpaired Student’s *t* test).

In addition, there is convincing evidence of immunosuppressive and tumorigenic roles in activated stromal and regulatory B cells, depending on the tumor subtype. These cells can prevent promotor activation involved in the antitumor immune response and significantly inhibit the efficacy of immunotherapy^25–27^. Similarly, PD-1 and PD-L2 were the only immune checkpoints that were expressed to a greater extent in Subtype B than Subtype A in both cohorts (LUAD cohort: *P*_*PD-1*_ = 3.99 × 10^−3^, *P*_*PD-L2*_ = 2.20 × 10^−7^ via unpaired Student’s *t* test; TCGA LUAD cohort: *P*_*PD-1*_ = 1.54 × 10^−4^, *P*_*PD-L2*_ = 1.51 × 10^−2^ via unpaired Student’s *t* test). Previous studies have confirmed that infiltrating regulatory B cells in lung cancer promote tumor growth and frequently stimulate the expression of immune checkpoints such as PD-L1 and PD1 by inhibiting T cell function^28,29^.

### The clinical relevance of LUAD subtypes

**Figure 4:**
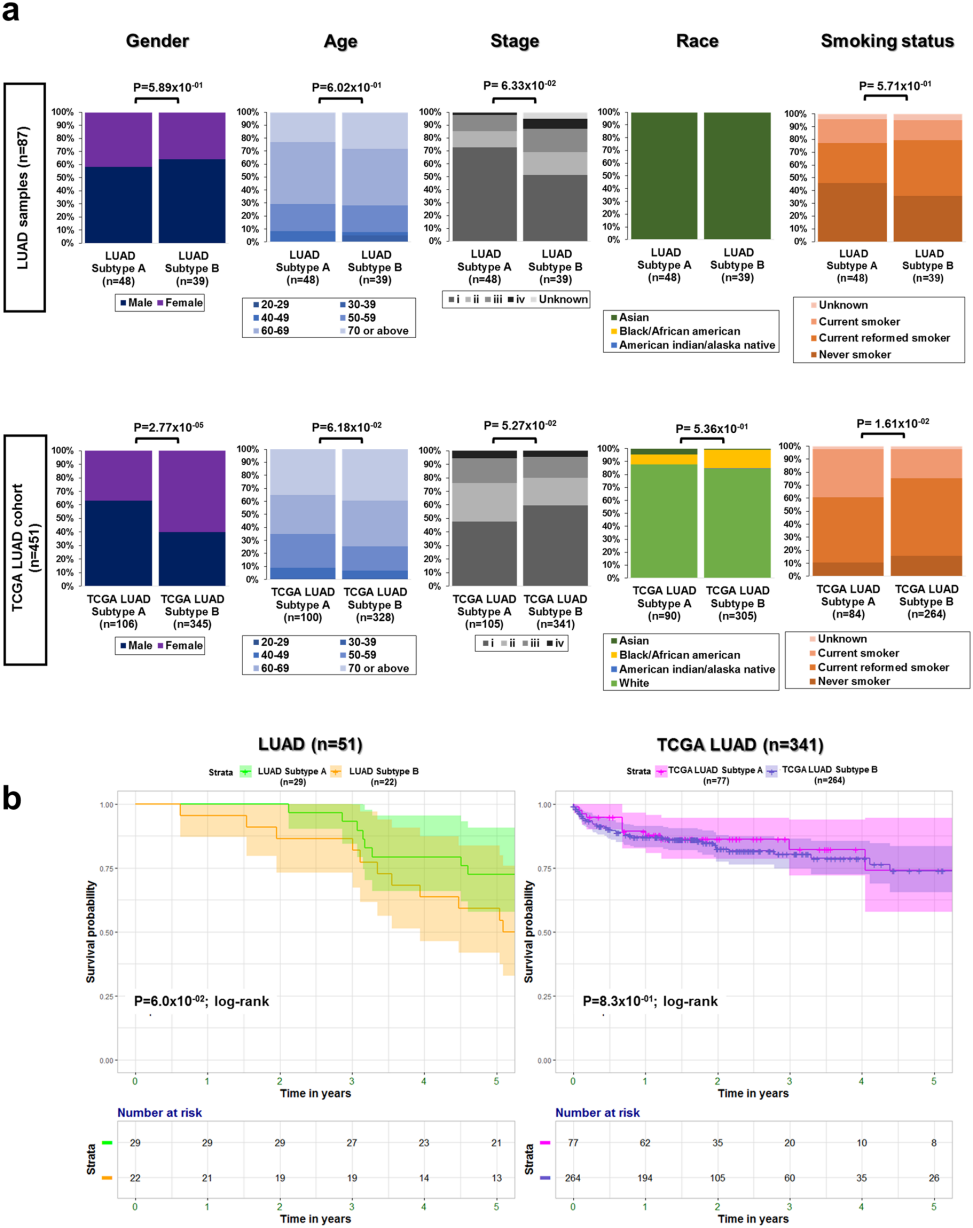
The clinical association in LUADs. (**a**) Distribution of gender, age, stage, race, and smoking status was analyzed between subtypes, and a p-value was indicated by an unpaired Student’s *t* test in LUAD and TCGA LUAD samples, respectively. (**b**) Survival curves and a risk table for LUAD (*n* = 51) and TCGA LUAD (*n* = 341) samples were visualized based on Kaplan-Meier estimates.

Demographic distributions of gender, age, stage, race, and smoking status were compared between patients with these cancer cell subtypes, who had been diagnosed with LUAD and TCGA LUAD. Only gender and smoking status elicited a significant difference between subtypes in the TCGA LUAD cohort (*P*_*gender*_ = 2.77 × 10^−05^, *P*_*smoking status*_ = 1.61 × 10^−02^ via the Mann Whitney U test; Fig. 4a).

Overall survival showed no significant difference between subtypes in both cohorts (*P*_*LUAD*_ = 6.0 × 10^−02^, *P*_*TCGA LUAD*_ = 8.3 × 10^−01^ via the log-rank test; Fig. 4b). Measured pathogenic tumor size was further compared between Subtypes A and B in LUAD patients (*n* = 51), revealing that the median tumor size in Subtype B was larger than that of Subtype A (Supplementary Fig. S8). Most likely, the frequency of infiltrating immune cells resulted in tumor progression, which increased tumor size^30^.

### Comparison of micro-environmental signatures between NSCLCs

**Figure 5:**
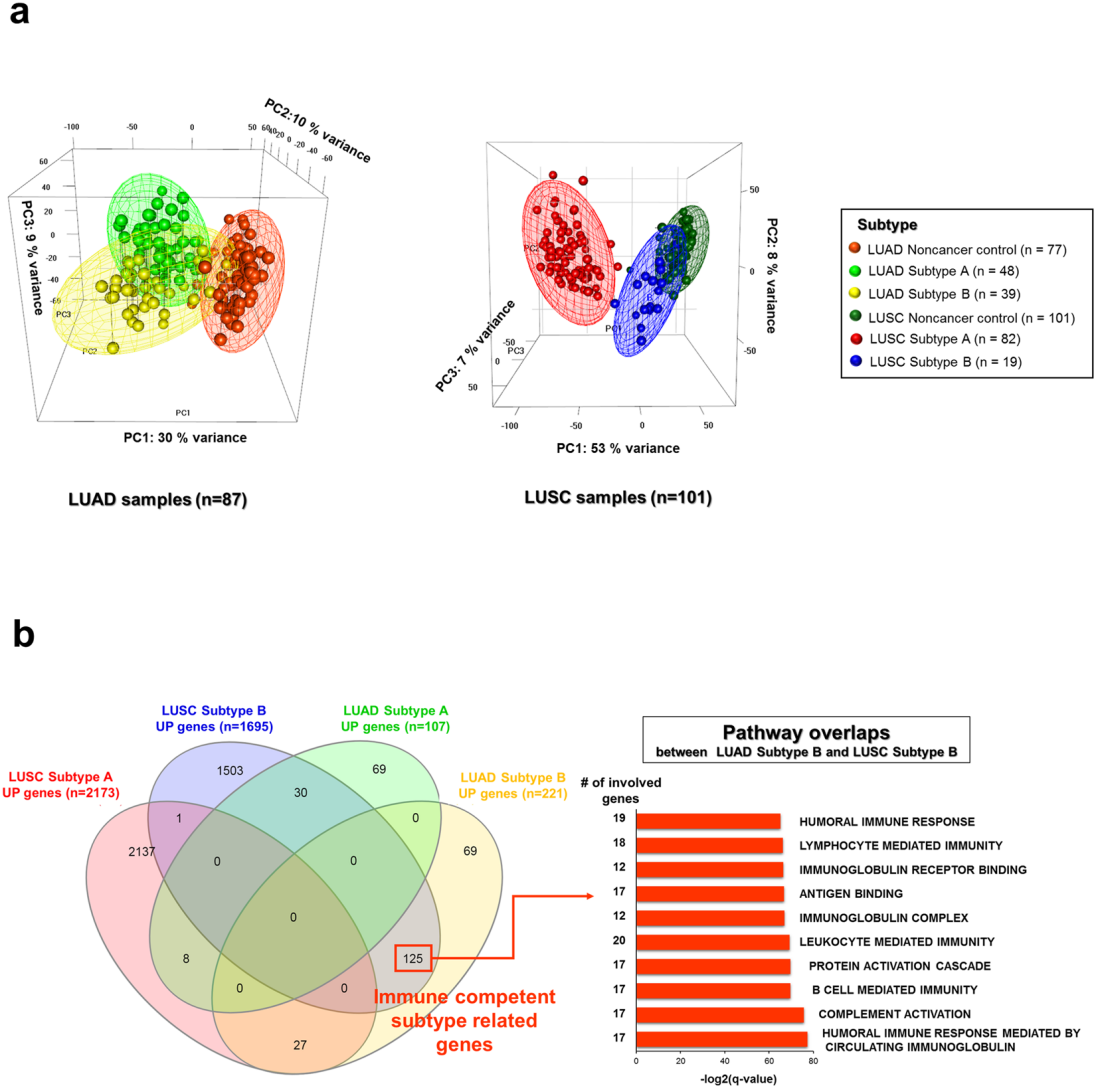
Comparison of micro-environmental signatures between subtypes in LUAD (*n* = 87) and LUSC (*n* = 101) samples. (**a**) PCA analysis for Subtypes A and B, and adjacent noncancer control samples in LUAD and LUSC samples. LUAD and adjacent noncancer control samples in LUADs and LUSCs were analyzed via the first three PCs of the top 1,000 most variable genes, and the three separated meshes of Subtypes A and B and noncancer control points with 95% confidence interval ellipsoids. (**b**) Venn diagrams were visualized with the shared genes for LUAD and LUSC subtype-specific up regulated genes, and shared top 10 GO gene sets between LUAD Subtype B and LUSC Subtype B.

PCA analyses of Subtypes A and B, as well as adjacent noncancer control samples for LUADs (*n* = 87) and LUSCs (*n* = 101), were performed on the first three principle components (PCs) of the 1,000 most variable genes. The three meshes of Subtypes A and B, as well as noncancer control points, were well-separated in both LUAD and LUSC samples (Fig. 5a). In both LUAD and LUSC cohorts, Subtype B overlapped with the noncancer control. LUSC Subtype B (*n* = 19) was more clearly distinguishable from Subtype A (*n* = 82) in the same sample type than Subtype B (*n* = 39) of LUAD samples, since the first PC (53% variance) of LUSC was higher than that of LUADs (30% variance).

Subtype B of LUSC samples was more closely associated with the noncancer control samples than Subtype A; whereas, Subtype B in LUAD samples was more closely associated with Subtype A in the same samples. The differentially expressed genes in each subtype were compared between LUSC and LUAD samples (Supplementary Table S1). The DEGs involved in each subtype were analyzed with respect to enrichment of Gene Ontology gene sets. There were 125 shared genes for upregulated expressed genes in LUAD and LUSC Subtype B; whereas, only eight genes were shared between LUAD and LUSC Subtype A (Fig. 5b).

The 125 shared genes between Subtype B in both cohorts were enriched in the humoral immune response as well as leukocyte and lymphocyte-mediated immunity. This result confirmed that Subtype B in LUADs and LUSCs can be categorized as an immune-competent subtype that signals many infiltrating lymphocytes and cytolytic activity, even in fundamentally different cancer types.

To compare micro-environmental factors between LUAD and LUSC subtypes, several generated micro-environmental factors (stromal, immune, cytolytic score, and tumor purity) were investigated by RNA expression data. All scores of micro-environmental factors were significantly higher in Subtype B for both LUAD and LUSC samples (Supplementary Fig. S9). In particular, the correlation between stromal and immune scores were compared between LUAD and LUSC subtypes, and it was confirmed that this correlation followed a different pattern according to cancer type (Supplementary Fig. S10).

For LUADs, the stromal score was more highly correlated with the immune score in both LUAD Subtype A (Pearson’s *r* = 0.86) and LUAD Subtype B (Pearson’s *r* = 0.86) than for the LUAD noncancer control (Pearson’s *r* = 0.35). Conversely, LUSC Subtype B (Pearson’s *r* = 0.46) had a lower correlation between these parameters than LUSC Subtype A (Pearson’s *r* = 0.79) and the noncancer control (Pearson’s *r* = 0.70). Furthermore, the data for LUSC Subtype B was densely aggregated in the high immune and stromal score ranges; whereas, that for LUAD Subtype B was widely scattered. These results affirm previous findings that the pattern of admixture between stromal and immune cells within tumor micro-environments results in changes to the pathogenesis of cancer and metabolism^31,32^.

The composition of stromal cells and their cytokine secretion in tumor micro-environments could distinctively impact the tumor progression and immune response in LUADs and LUSCs^33^. In addition, modulations to the micro-environment could take on a different pattern of admixture of stromal and immune cells depending on the immune and cancerous subtypes^34^.

**Figure 6:**
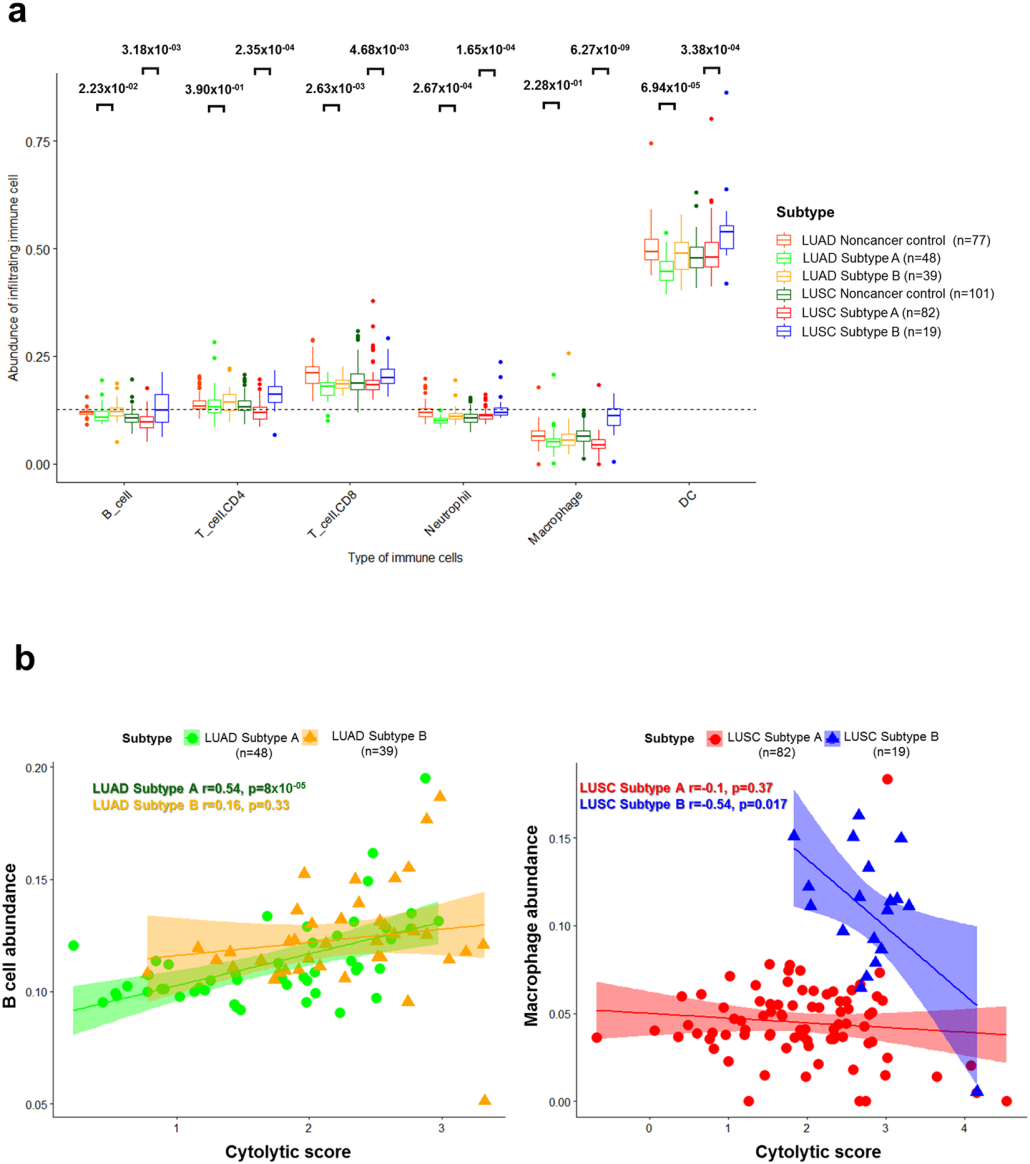
The distribution of infiltrating immune cells and the association with cytolytic score in NSCLCs. (**a**) The abundance of six types of infiltrating immune cells (B cells, CD4^+^ T cells, CD8^+^ T cells, neutrophils, macrophages, and dendritic cells) in LUAD and LUSC samples were estimated and compared between subtypes, and each p-value was indicated by an unpaired Student’s *t* test and the Mann-Whitney U test based on the sample distribution. (**b**) The scatter plots between cytolytic (CYT) score and immune cells (B cells for LUADs and macrophages for LUSCs) are illustrated with the Pearson’s correlation coefficient and p-value.

Similarly, the abundance of six types of infiltrating immune cells (B, CD4^+^ T, CD8^+^ T, neutrophils, macrophages, and dendritic cells) in LUAD and LUSC samples were estimated and compared between subtypes. All immune cells were more abundant in Subtype B than Subtype A (Fig. 6a). Interestingly, compared to LUSCs, macrophages and CD4^+^ T cells had no significant difference in population between subtypes in LUADs. Macrophages had the most detrimental impact on LUSC Subtype B, while B cells played this role in LUAD Subtype B.

### The impacts of micro-environment and immune checkpoints on NSCLCs

To ensure that B cells and macrophages had detrimental impacts on LUAD and LUSC Subtype B, correlations between six type of immune cells and micro-environmental factors were analyzed (Supplementary Fig. S11). Macrophages in LUSC Subtype B (Pearson’s *r* = −0.54) had a higher negative correlation with the cytolytic score compared to Subtype A (Pearson’s *r* = −0.1), while B cells in LUAD Subtype B (Pearson’s *r* = 0.16) had a lower correlation than Subtype A (Pearson’s *r* = 0.54; Fig. 6b).

Consistent with previous findings, it was found that B cells and macrophages had a low correlation with intra-tumoral immune cytolytic activity^35^, and correlations were further reduced in Subtype B than Subtype A in both LUADs and LUSCs. This indicated that the cytolytic activity upon CD8^+^ T cell activation as well as the efficacy of immune checkpoint blockade therapies were decreased by the abundance of B cells and macrophages in Subtype B, since the CD8^+^ T cell activation and immune checkpoint blockades had a more immediate and vital influence on cytolytic activity than B cells and macrophages^36^.

**Figure 7:**
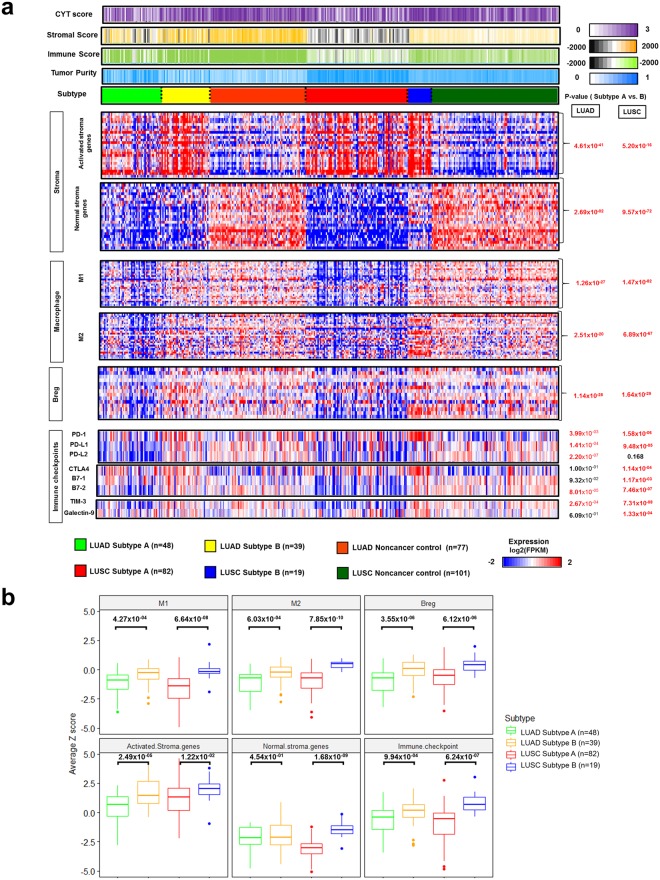
The impact of micro-environment and immune checkpoint expression on immune subtypes in NSCLCs. (**a**) The heatmap depicted the degree of the tumor micro-environmental factor (cytolytic (CYT) score, stromal score, immune score, and tumor purity), and the comparison of log_2_-transformed gene expression (log_2_(fpkm)) of the stromal (activated stroma and normal stroma), macrophage (M1 and M2), B reg, immune checkpoint genes was performed between LUAD and LUSC subtypes. A p-value was indicated by the Mann-Whitney U test and unpaired Student’s *t* test, based on the sample normality. (**b**) The average z score for M1, M2, B reg, activated and normal stromal genes, and immune checkpoints was computed and compared between LUAD and LUSC subtypes. The p-value was indicated by the Mann-Whitney U test and unpaired Student’s *t* test, based on the sample normality.

Through a comprehensive analysis of the NSCLC micro-environment, the activated and normal stromal genes, regulatory B cells, and macrophages 1 and 2 were over-expressed in LUAD and LUSC Subtype B and not in Subtype A (Fig. 7a). However, there was a gap in immune checkpoint expression between LUADs and LUSCs. Although both PD1 and PD-L1 expression was higher in Subtype B, PD-L2 expression was significantly higher in LUAD Subtype B only. Other immune checkpoints such as CTLA4, B7-1and2, Tim-2, and Galectin-9 were also over-expressed in LUSC Subtype B.

The z scores for signature genes in macrophages 1 and 2, activated and normal stroma, B_reg_, and immune checkpoints in Subtype B were significantly elevated in LUAD and LUSC samples (Fig. 7b), while that for all micro-environmental factors was higher in LUSC Subtype B than LUAD Subtype B. The z score for immune checkpoints and macrophage 2 were also far higher in LUSC Subtype B than in LUAD Subtype B. This finding confirmed that immune checkpoint expression was affected by subtype and cancer type, and an immense influence of tumor micro-environment was apparent in NSCLCs especially. Thus, determining these conditions for immune cells and tumor micro-environment is necessary for the success of immune checkpoint inhibitors in response to NSCLCs^37–39^.

The LUSC subtypes were also more strongly influenced by the tumor micro-environment as well as immune checkpoints than LUAD subtypes. It was confirmed by the previous finding that differential expression patterns of immune response related genes between LUAD and LUSC progression were more rapidly and strongly repressed in LUSCs than in LUAD as well as immune response promoting genes^40^. Therefore, one of the characteristics in LUAD could be the weak association with immune signature even in the immune competent subtype of LUAD than in LUSC. In addition, a strong association with smoking-associated gene alterations and somatic copy number variation might explain the higher micro-environmental influence in LUSCs^17,41^.

Overall, LUAD Subtype B (immune-competent; *n* = 39) and LUSC Subtype B (*n* = 19) shared obvious similarities and differences (Table 1). Gene expression-based clusters for dividing Subtypes A and B were more clearly separated in LUSCs than LUADs, and the percentage of Subtype Bs in LUADs was larger. LUSC Subtype B was most similar to the noncancer control groups, and the correlation pattern between stromal and immune scores was higher in LUAD Subtype B than in LUSC Subtype B. This indicated that the degree of admixture between stromal and immune cells was varied depending on the cancer type even if two different cancer tissue shared the same immune competent subtype^21^.

**Table 1:** The descriptions of gene expression-based immune subtypes of NSCLC.

Characteristics	Type of NSCLC	
	LUAD	LUSC
# of patients	87	101
Subtype A	48 (55.17%)	82 (81.19%)
Subtype B	39 (44.83%)	19 (18.81%)
Subtype clustering between A and B	Not a clear separation	A clear separation between subtypes
The description of subtype B cluster	High similarity to Subtype A	Very high similarity to noncancer control samples
The correlation between stromal and immune score		
Subtype A	High positive correlation (0.86)	High positive correlation (0.79)
Subtype B	High positive correlation (0.86)	Low positive correlation (0.46)
Impacts of stromal cells on subtype		
Subtype A	Moderate activated and low normal stromal cells	Moderate activated and low normal stromal cells
Subtype B	High activated and moderate normal stroma cells	High activated and high normal stromal cells
The influential immune cells to subtypes	B, CD8^+^ T, neutrophil, dendritic cells	B, CD4^+^ T,CD8^+^ T, neutrophil, macrophage, dendritic cells
The correlation between macrophage/B cells and cytolytic activity		
Subtype A	Weak correlation (0.08)/positive correlation (0.54)	Weak correlation (−0.1)/weak correlation (0.01)
Subtype B	Weak correlation (−0.02)/low correlation (0.16)	Negative correlation (−0.54)/weak correlation (0.05)
Clinical association with subtypes	Gender, Stage	None
The association with immune checkpoints	PD1-PD-L1/L2 only	Closely associated with all immune checkpoints

Subtype A = Immune deficient subtype.

Subtype B = Immune competent subtype.

Further, macrophages had no significant impact on LUAD subtypes, but did affect LUSCs. As evidence, PD-1 and PD-L1/L2 only had statistical differences in gene expression between subtypes in LUADs; whereas, all immune checkpoint expression was statistically different between subtypes in LUSCs. Therefore, identifying immune subtypes and assessing the fundamental differences in micro-environmental signatures of NSCLCs were essential for understanding the state of stromal and immune cells in lung cancer and selecting the appropriate immune checkpoint blockades to observe, depending on subtype and cancer type. These observations are important for predicting potential immunotherapy responses.

## Discussion

Although the previously established biomarkers for predicting clinical outcomes of immunotherapy, such as PD1/PD-L1 expression, have not been a guarantee of success for all cancer patients, it is still important to find the most accurate and generalized predictive signatures in each patient^42^. Somatic mutations in coding regions, as well as mutations and neoantigen burden, have been influential factors in the efficacy of immunotherapy, and it has been demonstrated that immunogenic gene expression has been correlated with the type and extent of responses to immunotherapies^43,44^. Therefore, gene expression can be utilized to estimate the impact of immune subtypes and tumor micro-environment on the efficacy of immunotherapy in NSCLCs. Especially, quantifying tumor-infiltrating lymphocytes should be considered as a method of increasing response rates in patients^45,46^.

In this study, PCA analysis and hierarchical clustering based on the variance in gene expression were useful tools in determining the immune-deficient and competent subtypes in NSCLCs. The properties of defined immune subtypes were finally described by gene enrichment analysis with differentially expressed genes and tumor micro-environment factors, as well as clinical association.

Through a comprehensive analysis of tumor micro-environments, we showed that recruited tumor-associated stromal cells, such as activated and normal stromal cells as well as immune cells, in LUADs and LUSCs affect the tumor micro-environment and control tumor progression within immune-competent subtypes. This result is in accordance with reported tumor-associated stromal cells that play critical roles in the development of the tumor micro-environment, tumor angiogenesis, invasion, and therapeutic resistance^23^.

On the other hand, the impacts of tumor micro-environment on NSCLCs were varied depending on the specific immune subtype and cancer type, even if patients were grouped with the identical immune subtype in LUADs and LUSCs. Here, the degree of admixture between stromal and immune cells engendered significant differences in the tumor micro-environment.

Our results suggest that the immunosuppressive role and tumorigenesis of tumor-associated macrophages and regulatory B cells in the immune-competent subtype of NSCLCs could prevent promotion from the antitumor immune response and stimulate the expression of immune checkpoints such as PD-L1 and PD1 by inhibiting CD8^+^ T cell activation. The prevalence of these cells could reduce the effectiveness of immune checkpoint blockades in NSCLC patients. Therefore, understanding these cells and their interactions with immune checkpoints could help to treat NSCLC patients with immunotherapy successfully.

In conclusion, this work demonstrated that our computational methodologies for immune subtyping using gene expression patterning could be utilized to identify NSCLC patients who will be affected by tumor micro-environments and immune checkpoints. Therefore, characterizing recruited immune and stromal cells should help in identifying the prognostic and predictive factors that could guide a personalized approach to cancer immunotherapy. Additionally, understanding the state of stromal and immune cells in lung cancer, and identifying fundamental tumor micro-environment factors that impact cancer metabolism and immunity will give clinicians significant predictive power with respect to patient receptiveness to immunotherapies. Considering the immune subtypes and tumor micro-environment is a better target for predicting responses to immune therapy and is applicable for all cancer types. Future studies should seek to clinically and experimentally validate RNA expression-based immune subtypes by measuring immune cell populations in patients.

## Methods

### Sample Data Sets

The LUAD and LUSC RNA sequencing data as well as the matched adjacent noncancer control data–which were previously published by Seo *et al*.^8,17^–were used to analyze tumor micro-environments and immune subtypes for NSCLCs. LUAD and LUSC expression datasets (htseq count value) in The Cancer Genome Atlas (TCGA) were included for validation of our results.

### RNA-seq pre-processing

The pre-processed data from raw reads to htseq count was prepared by previously reported methods^17^. The RNA-seq reads were mapped to GRCh37 via the spliced transcripts alignment to a reference aligner, and the data processing steps on the GTAK website were followed for our data^47^. The number of raw reads was generated from the pre-processed data via HTSeq count for Ensembl-annotated genes, and the raw read expression values were transformed to variance-transformed data(VSD) (R package ‘DESeq2’). The HTseq count values were converted to fragments per kilobase million (FPKM) using the R package’edgeR’, and the expression values of both raw reads and FPKM were adjusted to median-centered and log2 gene expression (Cluster 3.0).

The library preparation for all our LUAD and LUSC samples was prepared in the same batches, and batch effect adjustment was not required^48^. However, TCGA data cannot be grouped with our LUAD and LUSC samples, since library preparation for the samples and human reference sources, as well as options for HT-seq counts for computing RNA expression values in our and TCGA samples, were totally different, and unknown batch effects also existed in RNA-seq data. Therefore, it was difficult to remove the batch effects between our LUAD and TCGA LUAD samples. Thus, we analyzed them separately and the results from TCGA LUAD samples were used for the validation of our LUAD sample subtypes because of the low quality reads in several LUAD samples, which made classification of the TCGA LUAD samples harder^49^.

### Unsupervised immune subtyping and differentially expressed gene (DEG)

Immune subtyping was performed based on a PCA analysis using the 1,000 most variable genes within all tumor and noncancer control samples. Hierarchical clustering and k-means (*n* = 3) based on the principal components were clustered to three different groups: cancer only, cancer and noncancer control, and mixed clusters using the R package ‘rgl’^50,51^. Based on such clustering, the subtypes were defined and the samples within each subtypes were plotted between each of the three highest PCA components, with a 95% confidence interval.

DEGs of Subtype B were compared to those of Subtype A, and the adjusted p value was estimated by previously reported methods and criteria (adjusted *P* < 0.05, |Log2 (fold change)| ≥ 1, and base mean ≥ 100)^17^. The expression of DEGs was visualized by a heatmap using JAVA treeview. The DEGs (Subtype A-UP and B-DOWN, Subtype A-DOWN and B-UP) were enriched for Gene Ontology (GO) gene sets via the web version of the Gene Set Enrichment Analysis (GSEA), and the top ten GO sets were indicated by the bar graph.

### Estimation of micro-environmental factors and distribution of infiltrating immune cells

Several micro-environmental factors (stromal, immune, and tumor purity) were generated by previously reported methods using the ESTIMATE algorithm^20^. The, CYT score and abundance of six types of infiltrating immune cells (B cells, CD4^+^ T cells, CD8^+^ T cells, neutrophils, macrophages, and dendritic cells) were estimated via the TIMER algorithm and compared between subtypes in LUAD and LUSC cohorts^21^.

### The signature genes in stromal, macrophage 2, and regulatory B cells

The expression (FPKM) of signature genes in stromal, macrophage 2, and regulatory B cells from previously validated gene sets was adjusted to median-centered and log_2_ transformed via cluster 3.0, then visualized by a heatmap (JAVA treeview)^52,53^.

### Calculation of z scores for micro-environmental factors

The z scores for signature genes in macrophages 1 and 2, activated and normal stroma, B_reg_, and immune checkpoints were calculated from log_2_ transformed and median-centered FPKM expression values, and the average z score for each factor was computed and compared between LUAD and LUSC subtypes^54^.

$$\begin{array}{cc}Z-score & =\frac{Expression\,in\,tumor\,samples\,(FPKM)-Mean\,expression\,in\,noncancer\,control\,samples\,(FPKM)}{standard\,deviation\,of\,expression\,in\,noncancer\,control\,samples\,(FPKM)}\end{array}$$

### Statistical test

Statistical analyses were performed using R-3.3.0. The p-value was computed based on the sample distribution, using the Shapiro-Wilk normality test. Comparisons between subtypes were analyzed using the unpaired Student’s *t* test or Mann-Whitney U test. Comparisons among more than two subgroups were analyzed using the Kruskal-Wallis or one-way ANOVA test. The correlation coefficient (*r*) was calculated via the Pearson’s coefficient and distance correlation methods. The overall survival curves and risk tables were visualized based on Kaplan-Meier estimates, using the R package ‘survminer’. The p-value was computed via a log-rank test.

## Electronic supplementary material

Supplementary Figures S1-S11

Supplementary Table S1

## Data Availability

LUSC and LUAD transcriptome sequencing data was uploaded to public databases. LUSC transcriptome sequencing data are available under the NCBI Sequence Read Archive accessions (no. SRP114315), and LUAD transcriptome sequencing data are available under the EBI European Nucleotide Archive accessions (no. ERP001058).
