# Supplementary material for: Comprehensive analysis of the tumor immune micro-environment in non-small cell lung cancer for efficacy of checkpoint inhibitor
Source: Sci Rep. 2018 Oct 1;8:14576. doi: 10.1038/s41598-018-32855-8 (PMC6167371; doi:10.1038/s41598-018-32855-8)
Supplement: Supplementary file 1 — Supplementary Figures S1-S11 [file 41598_2018_32855_MOESM1_ESM.pdf]

Comprehensive analysis of the tumor immune micro-environment in non-small cell lung cancer for efficacy of checkpoint inhibitor

Jeong-Sun Seo[1,2,3,6,7,*], Ahreum Kim[1,2,3,7], Jong-Yeon Shin[2,6] & Young Tae Kim[2,4,5,*]

Supplementary Figure S1. PCA analysis for LUAD and adjacent noncancer control samples.

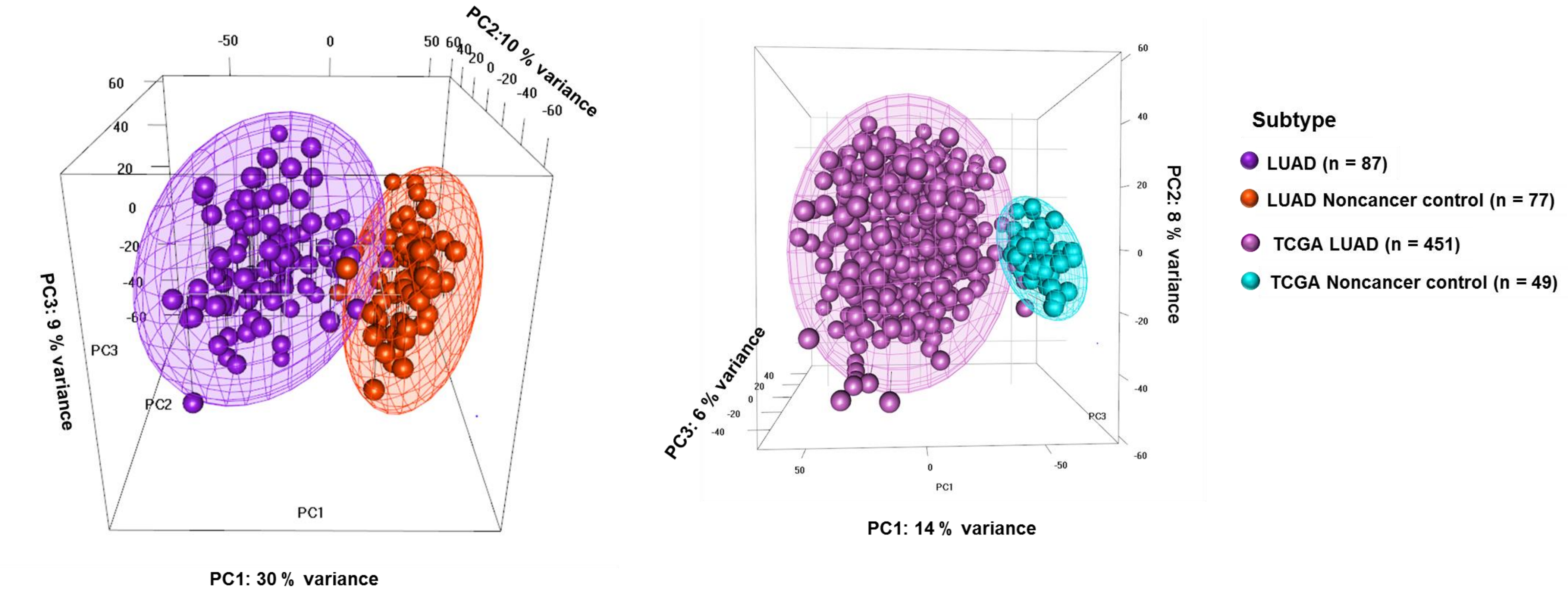

LUAD samples (n=87)

TCGA LUAD cohort (n=451)

LUADs and adjacent noncancer control samples in LUAD(n=87) and TCGA LUAD (n=451) were analyzed by the first three PCs of the top 1,000 most variable genes. The meshes containing each LUAD and noncancer control points were drawn by performing unsupervised hierarchical clustering and k-means clustering (k-means = 2), with 95 % confidence interval ellipsoids in LUAD and TCGA LUAD samples.

Supplementary Figure S2. Identification of two distinct molecular subtypes in LUAD and TCGA LUAD samples.

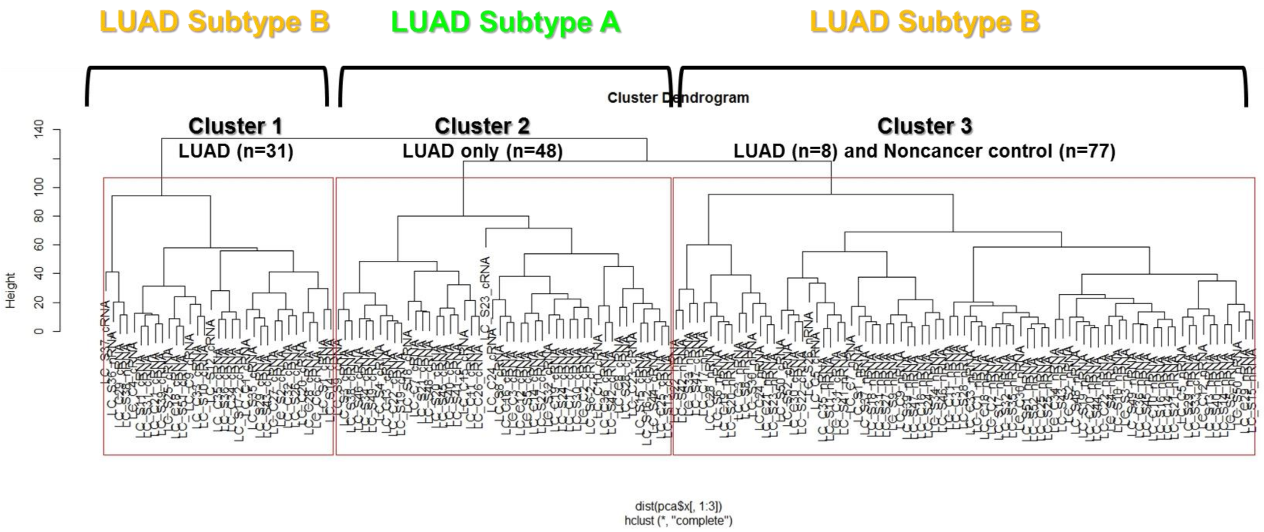

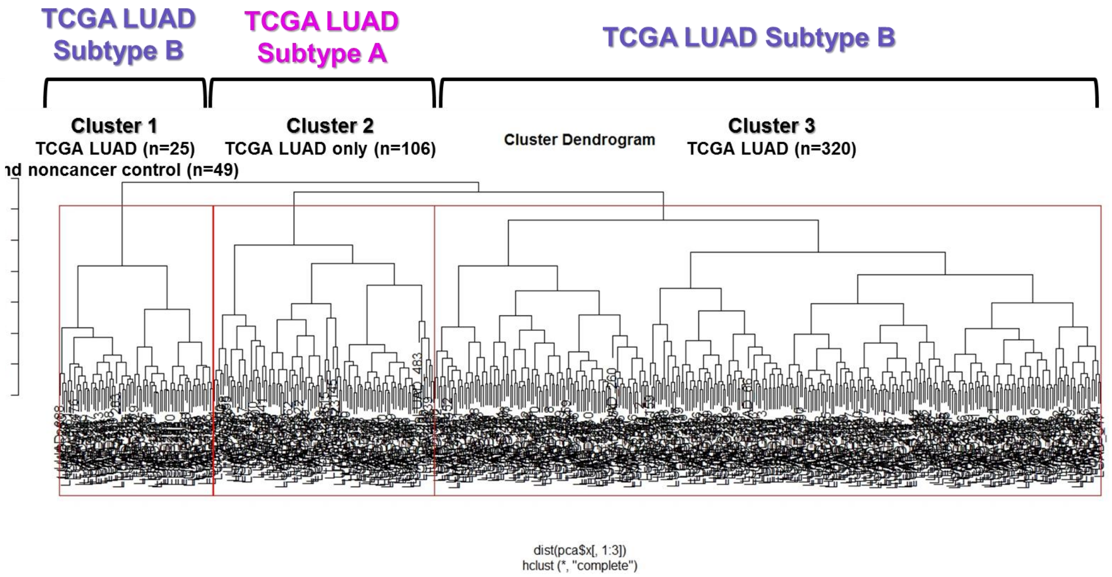

The unsupervised hierarchical clustering and k-means clustering (k-means = 3) were performed by the first three PCs of the top 1,000 most variable genes in LUAD(n=87) and TCGA LUAD (n=451) samples, and the two distinct molecular subtypes on LUADs and TCGA LUADs were defined as Subtype A and Subtype B.

Supplementary Figure S3. Transcriptomic analyses for immune subtypes in TCGA LUADs.

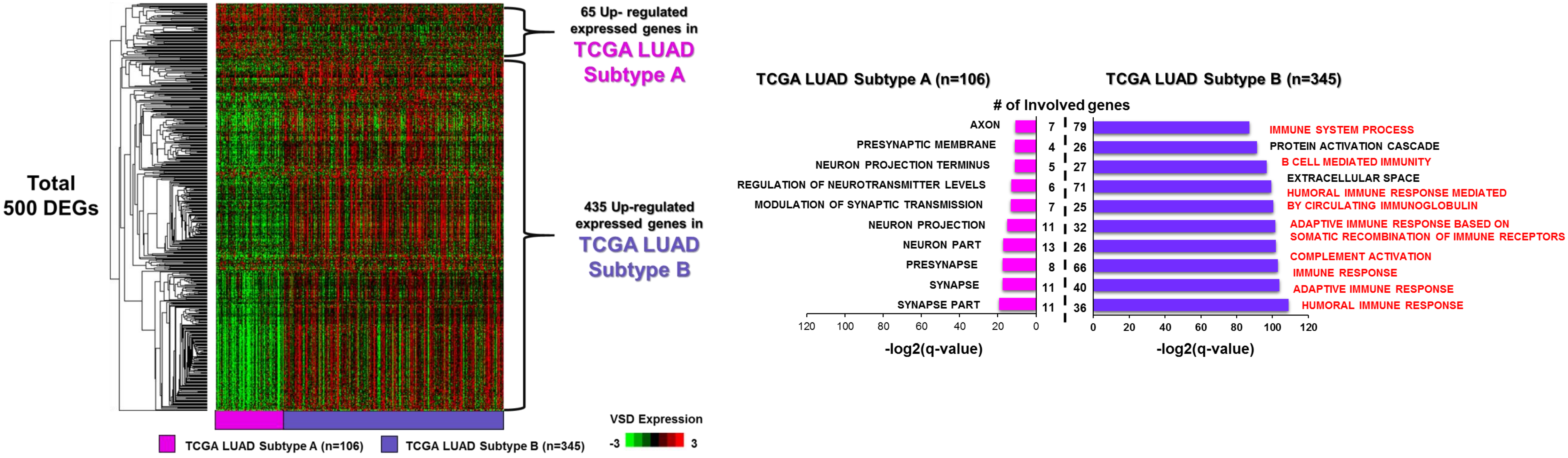

The VSD-normalized expression of differentially expressed genes in Subtypes A and B of TCGA LUADs are illustrated in the heatmap. Top 10 GO gene sets in either Subtype A or B were selected based on the rank of enrichment –log2(q-value) for the pathway and matched significance criteria (p-value <0.05 and FDR q-value < 0.1). A two-color scale was used, with green indicating low expression values and red representing highly expressed genes.

Supplementary Figure S4. Venn diagrams for shared genes between LUAD and TCGA LUAD immune subtypes.

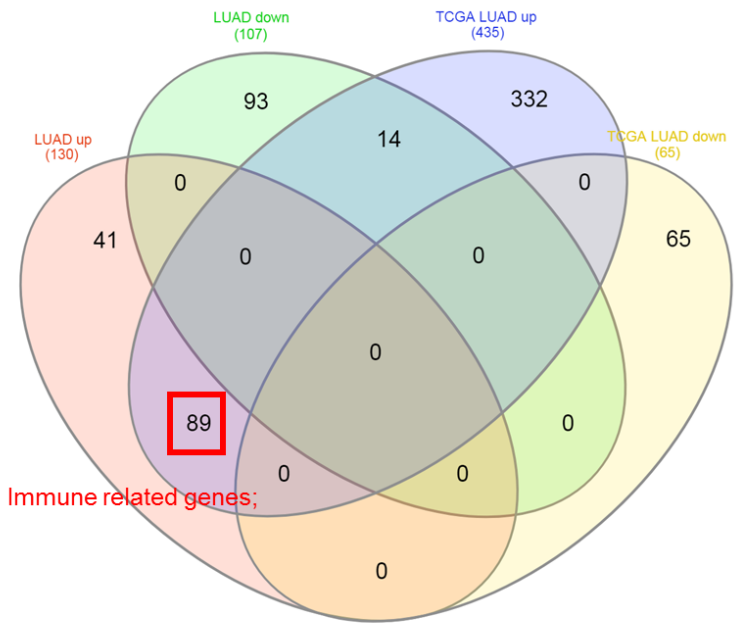

PATHWAYS	# Genes in Overlap (k)	FDR q-value
HUMORAL IMMUNE RESPONSE MEDIATED BY CIRCULATING IMMUNOGLOBULIN	16	6.58E-26
COMPLEMENT ACTIVATION	16	1.84E-25
B CELL MEDIATED IMMUNITY	16	9.30E-24
PROTEIN ACTIVATION CASCADE	16	9.30E-24
ANTIGEN BINDING	16	8.31E-23
HUMORAL IMMUNE RESPONSE	17	3.70E-21
IMMUNOGLOBULIN COMPLEX	11	3.70E-21
LYMPHOCYTE MEDIATED IMMUNITY	16	3.74E-21
IMMUNOGLOBULIN RECEPTOR BINDING	11	4.85E-21
ADAPTIVE IMMUNE RESPONSE BASED ON SOMATIC RECOMBINATION OF IMMUNE RECEPTORS BUILT FROM IMMUNOGLOBULIN SUPERFAMILY DOMAINS	16	6.49E-21

Venn diagrams was visualized by the shared genes for LUAD and TCGA LUAD subtype- specific up regulated genes and shared top 10 GO gene sets between LUAD Subtype B and TCGA LUAD Subtype B.

Supplementary Figure S5. The comparison of correlation between stromal and immune scores in LUAD and TCGA LUAD immune subtypes.

Subtype	P-value	Correlation coefficient (r)	
	Pearson's	Pearson's	Distance
LUAD Noncancer control (n=77)	1.52×10^{-03}	0.355	0.374
LUAD Subtype A (n=48)	6.26×10^{-15}	0.858	0.860
LUAD Subtype B (n=39)	3.70×10^{-12}	0.856	0.819
TCGA LUAD Noncancer control (n=49)	2.30×10^{-02}	0.324	0.348
TCGA LUAD Subtype A (n=106)	2.37×10^{-20}	0.750	0.706
TCGA LUAD Subtype B (n=345)	6.46×10^{-42}	0.645	0.591

The Pearson's and distance correlation coefficients between stromal and immune scores were computed and represented in a chart for each of the subtypes and noncancer controls in LUAD and TCGA LUAD samples.

Supplementary Figure S6. Estimation of distribution of infiltrating immune cells between subtypes in LUADs and TCGA LUADs.

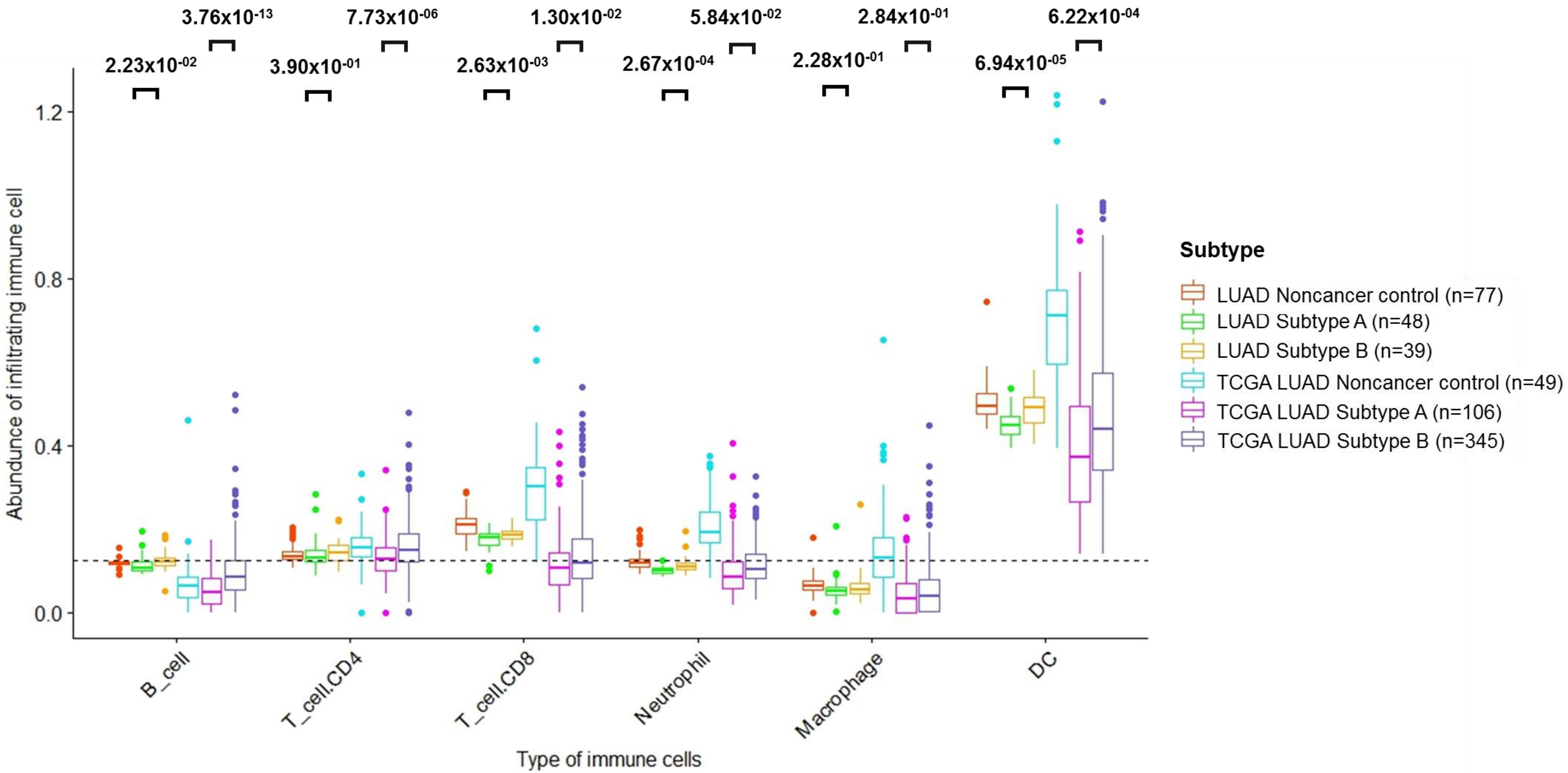

The abundance of six types of infiltrating immune cells (B cells, $CD4^+$ T cells, $CD8^+$ T cells, neutrophils, macrophages, and dendritic cells) in LUAD and LUAD TCGA samples were estimated and compared between subtypes, and each p-value was indicated based on the sample distribution. The median of immune cell abundance is indicated by a horizontal dashed line.

Supplementary Figure S7. The correlation of infiltrating immune cells and micro-environmental factors in LUAD and TCGA LUAD immune subtypes.

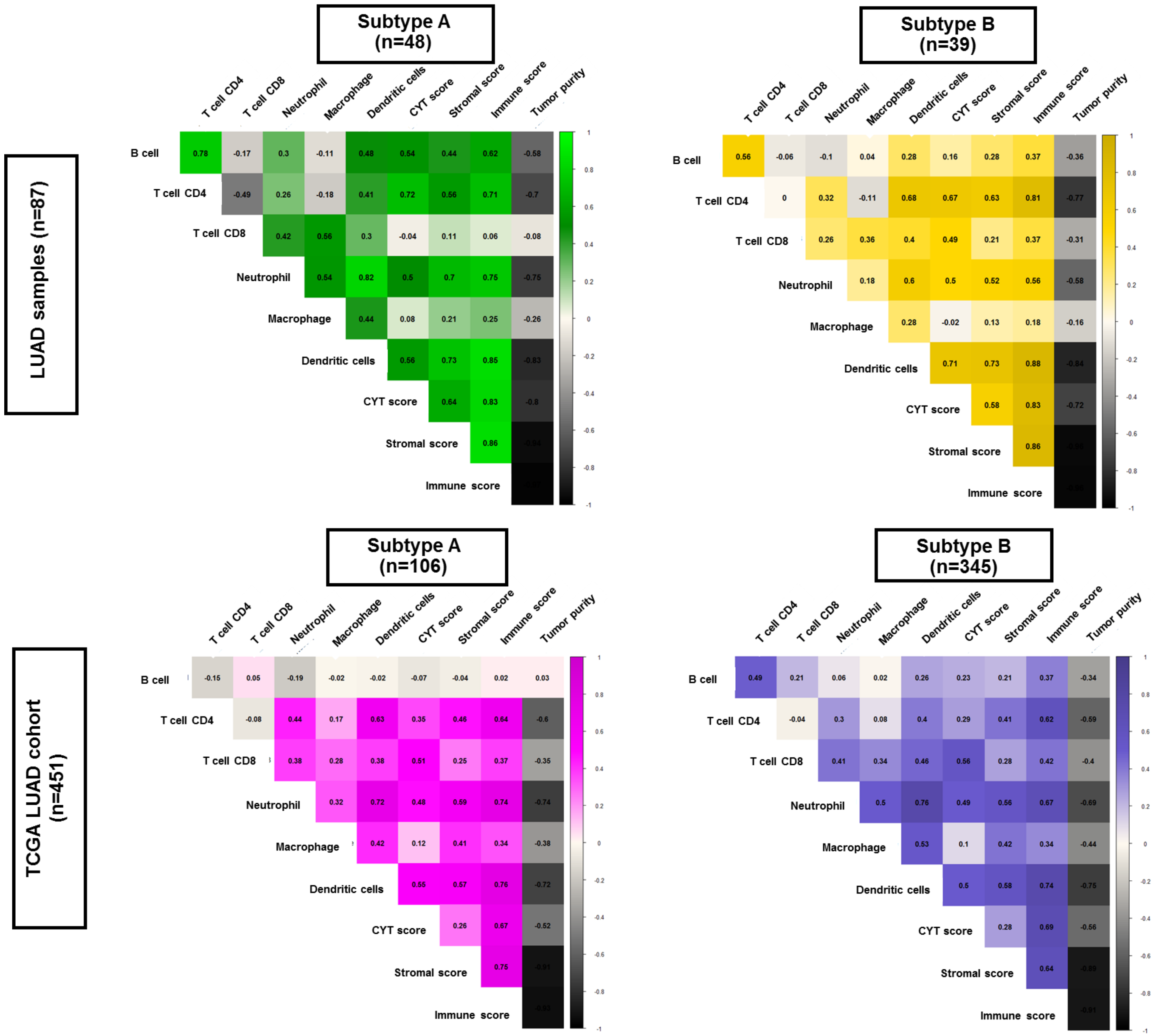

The heat map indicated the correlation between six type of immune cells and micro-environmental factors in Subtype A and B of LUAD and TCGA LUAD samples, respectively. A five color scale was used with green (LUAD Subtype A), yellow (LUAD Subtype B), magenta (TCGA LUAD Subtype A), slateblue (TCGA LUAD Subtype B) indicating high correlation values and black representing low correlation values.

Supplementary Figure S8. The comparison of pTsize between subtypes in LUADs (n=51).

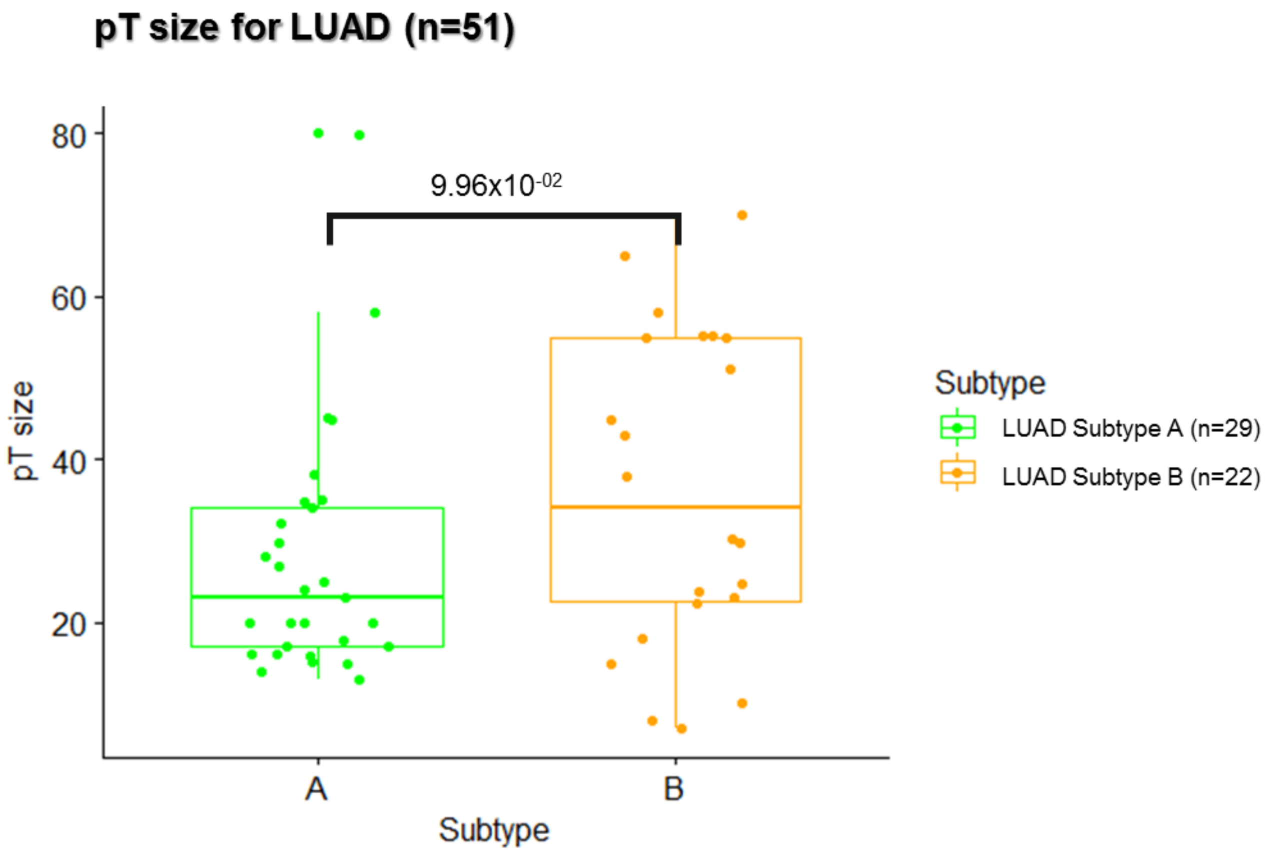

The measured pTsize was compared between Subtype A and B in LUADs (n=51) with indication of Mann-Whitney p-value.

Supplementary Figure S9. The comparison of micro-environmental factors between subtypes in LUAD and LUSC.

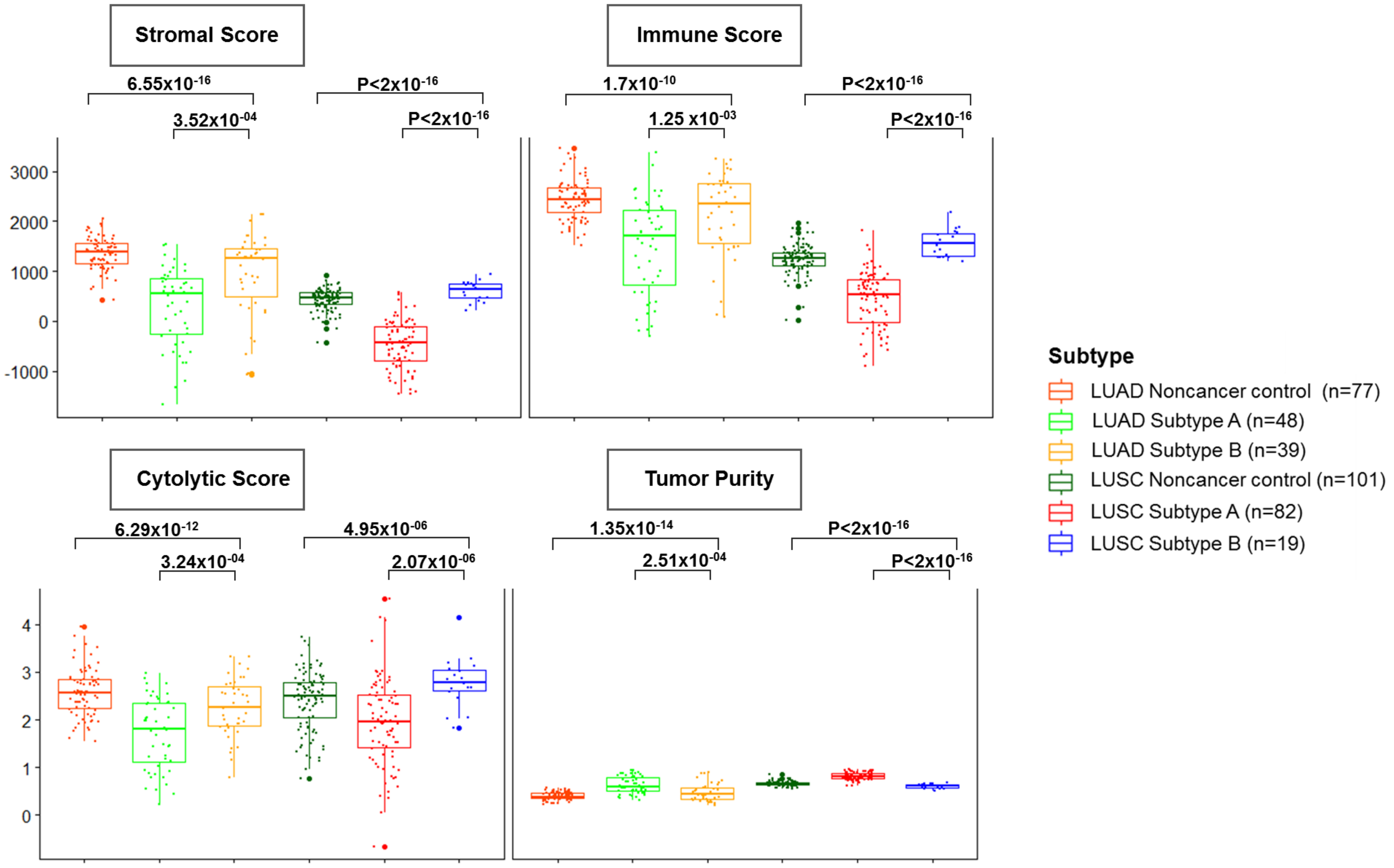

Several generated micro-environmental factors (stromal, immune, cytolytic (CYT) score, and tumor purity) were compared between subtypes in LUAD and LUSC samples, and the each p-value was indicated by statistical analysis based on the sample distribution test.

Supplementary Figure S10. The comparison of correlation between stromal and immune scores in LUAD and LUSC immune subtypes.

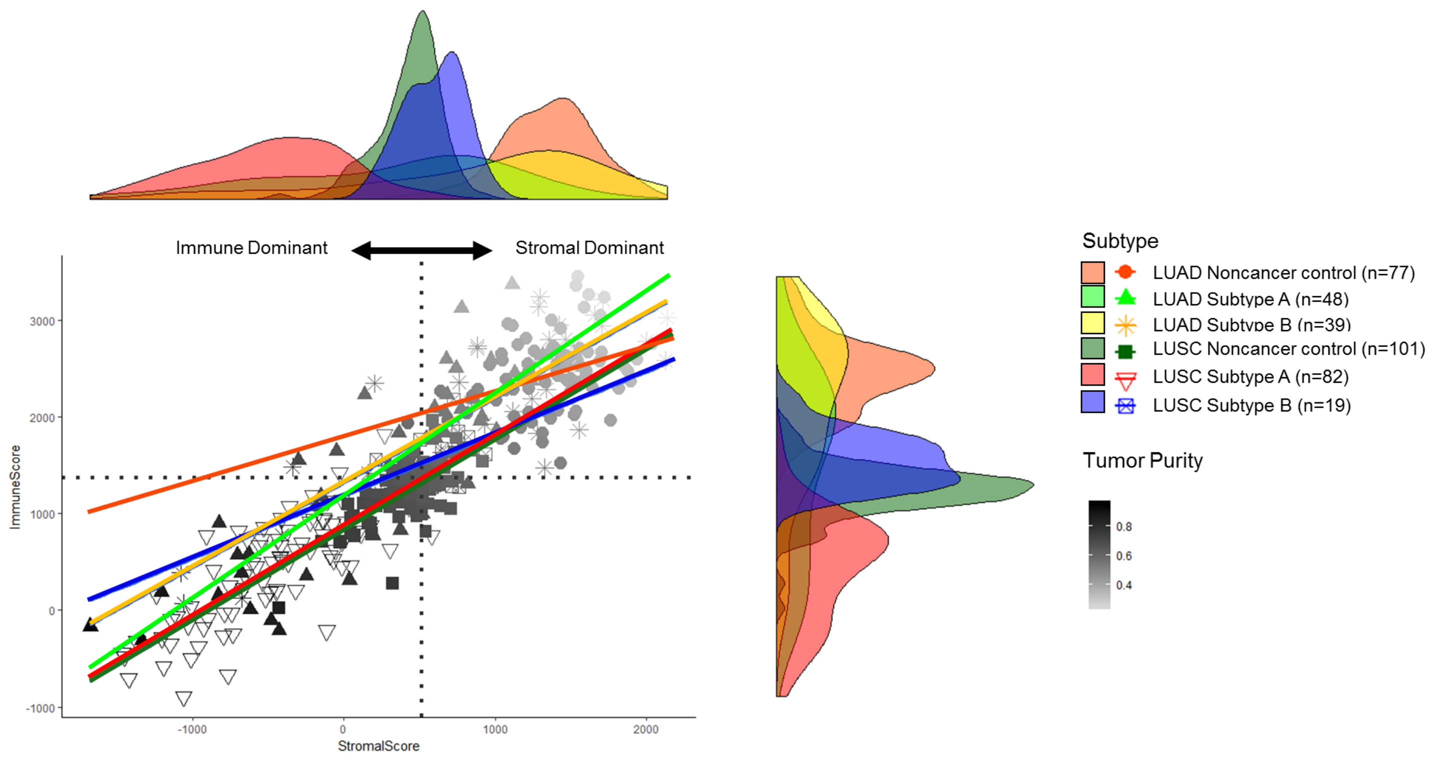

**Subtype**	**P-value**	**Correlation coefficient (r)**	
	**Pearson's**	**Pearson's**	**Distance**
**LUAD Noncancer control (n=77)**	$1.52x10^{-03}$	0.355	0.374
**LUAD Subtype A (n=48)**	$6.26x10^{-15}$	0.858	0.860
**LUAD Subtype B (n=39)**	$3.70x10^{-12}$	0.856	0.819
**LUSC Noncancer control (n=101)**	$2.11x10^{-16}$	0.704	0.653
**LUSC Subtype A (n=82)**	$1.14x10^{-18}$	0.790	0.784
**LUSC Subtype B (n=19)**	$4.56x10^{-02}$	0.464	0.539

Scatterplots along with marginal density plots between stromal and immune scores were illustrated, and the tumor purity was used as the color grading of sample points, and its index was shown on the color bar at the bottom right of the plot. The Pearson’s and distance correlation coefficient were computed and represented in a chart by each of the subtypes and normal in LUAD and LUSC samples.

Supplementary Figure S11. The correlation of micro-environmental factors and distribution of infiltrating immune cells between subtypes in LUADs and LUSCs.

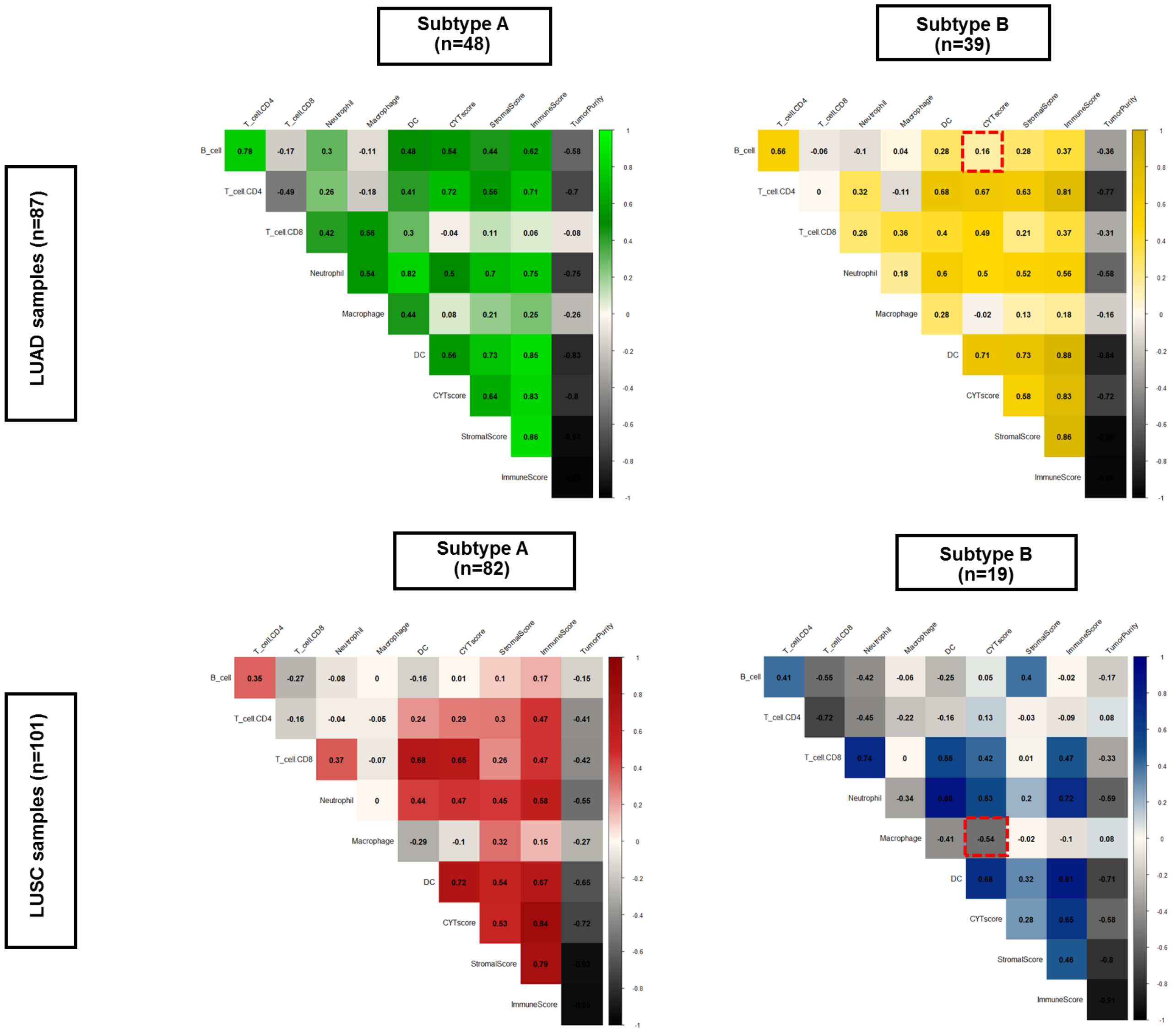

The correlation between six type of immune cells and micro-environmental factors in LUAD and LUSC subtypes was indicated by a heatmap. A five color scale was used with green, yellow, red and blue indicating high correlation values and dark grey representing low correlation values. The immune cell which gives rise to negative impact on CYT score in subtype B was indicated by red dot box.
